# Rapid Decision Algorithm for Patient Triage during Ebola Outbreaks

**DOI:** 10.3201/eid3011.231650

**Published:** 2024-11

**Authors:** Denis-Luc Ardiet, Justus Nsio, Gaston Komanda, Rebecca M. Coulborn, Emmanuel Grellety, Francesco Grandesso, Richard Kitenge, Dolla L. Ngwanga, Bibiche Matady, Guyguy Manangama, Mathias Mossoko, John K. Ngwama, Placide Mbala, Francisco Luquero, Klaudia Porten, Steve Ahuka-Mundeke

**Affiliations:** Epicentre, Paris, France (D.-L. Ardiet, G. Komanda, R.M. Coulborn, E. Grellety, F. Grandesso, F. Luquero, K. Porten); Ministry of Health, Kinshasa, Democratic Republic of the Congo (J. Nsio, R. Kitenge, D.L. Ngwanga, B. Matady, M. Mossoko, J.K. Ngwama); Médecins Sans Frontières France, Paris (G. Manangama); Institut National de la Recherche Biomédicale, Kinshasa (P. Mbala, S. Ahuka-Mundeke); University of Kinshasa, Kinshasa (S. Ahuka-Mundeke)

**Keywords:** Ebola, Algorithm, triage, outbreaks, exposure, contact, clinical signs, symptoms, symptomatology, predictors, epidemics, Democratic Republic of the Congo

## Abstract

The low specificity of Ebola virus disease clinical signs increases the risk for nosocomial transmission to patients and healthcare workers during outbreaks. Reducing this risk requires identifying patients with a high likelihood of Ebola virus infection. Analyses of retrospective data from patients suspected of having Ebola virus infection identified 13 strong predictors and time from disease onset as constituents of a prediction score for Ebola virus disease. We also noted 4 highly predictive variables that could distinguish patients at high risk for infection, independent of their scores. External validation of this algorithm on retrospective data revealed the probability of infection continuously increased with the score.

Suspicion of Ebola virus disease (EVD) based solely on clinical grounds constitutes a challenge for healthcare workers, given nonspecific symptomatology, especially in early phases, which can be associated with other acute infections (*1*). Consequently, reducing the risk for transmission to healthcare workers, patients, or caretakers (*2**,**3*) requires the use of broad case definitions for EVD suspicion (*4**-**8*) and use of systematic isolation and testing, even if only a small proportion of possible cases are likely infected. This diagnostic approach requires molecular diagnosis (reverse transcription PCR [RT-PCR]) for large numbers of samples. Drawbacks of this strategy include high costs and overcrowding of isolation wards (*9**,**10*), delayed consideration of other serious diseases (*11*), and dissatisfaction with and nonadherence to response measures (*12*), all of which can lead to community transmission.

The ability to classify patients rapidly and reliably in terms of their probability of receiving a confirmed diagnosis of EVD could potentially improve response efficiency and acceptance. Such classification might inform standard isolation measures and reprioritize RT-PCR testing for suspected cases, shifting focus to a smaller, high-risk category of patients, reducing nosocomial transmission in health centers and Ebola isolation units (*13*). This type of triaged approach might not only expedite diagnosis, ultimately improving management and outcome of EVD-positive patients (*14*), but also help control outbreaks through faster identification of case patients and downstream contacts. Identifying early EVD cases through this means of classification might also enable more rapid detection of the illness in healthcare facilities, before outbreak declaration.

Clinical decision algorithms and scoring-based tools aim to evaluate the probability of infection or of severity of a disease based on clinical and epidemiologic evidence and can be used to screen and classify patients before diagnosis or treatment. Regarding Ebola suspicion, previous publications have derived scores on the basis of predictors, producing calculated prediction scores for Ebola infection (*6**,**15**-**21*). Most of those algorithms, however, were developed from small datasets and lack prospective validation. Nonetheless, some reports suggest that the development of prediction scores might hold promise as EVD risk classification tools.

Our previous work identified 2 disease phases of EVD, with distinct clinical manifestations (*22*). On the basis of that data, we developed and evaluated a new, rapid-decision algorithm to assess EVD risk. Comprised of 4 priority variables, 13 scoring variables, and time from symptom onset to seeking care (referred to as time-to-presentation in this article), this algorithm seeks to define 3 categories of Ebola infection risk: low-risk, intermediate-risk, and high-risk.

## Methods

### Study Population, Datasets, and Variables

The training dataset, described in detail elsewhere (*22*), encompasses all patients suspected of having EVD (EVD-suspected patients) in Ituri and North Kivu provinces, Democratic Republic of the Congo (DRC). Data were collected during August 1, 2018–August 28, 2019, from 30 different Ebola treatment or transit centers and small, decentralized isolation units. Variables of interest for prediction of infection included time-to-presentation, age, 34 clinical variables, final GeneXpert (Cepheid, https://www.cepheid.com) RT-PCR status, and 4 possible exposure histories: contact with a known EVD-positive person, attendance at any funeral, health facility consultation for any reason, and consultation with an informal health practitioner for any reason. Following methods previously described (*22*), we considered 2 patient groups based on time-to-presentation (short vs. long), separated by a threshold between day 2 and day 3 after symptom onset (with symptom onset self-reported by patients or their relatives during in-depth epidemiologic investigations by trained investigators and clinical teams). Newly EVD-suspected cases occurring during August 15–November 28, 2019, comprised the testing dataset, which consisted of 14,346 patients, among whom 319 (2.2%) were confirmed EVD-positive by RT-PCR (Appendix).

### Development of the Triage Algorithm

The diagnostic performance of predictors is reported from the training dataset according to multivariate logistic regression diagnostics (*22*), enabling a first selection of main predictors. The association of predictors with infection was either positive or negative and could vary by time-to-presentation. We incorporated 2 components into the algorithm: a prioritization rule for variables highly predictive of infection, and an EVD prediction score based on other variables having strong positive or negative associations with infection, also considering time-to-presentation. For this second component, we calculated individual scores from the regression diagnostics, based on β coefficients. We used a variable selection process to evaluate the performance of different sets of predictors with different ranges of individual scores. We evaluated versions of the algorithm on 30 bootstrapped samples of the training dataset and compared classification performance (area under the receiver operating characteristic [AUROC] curve). Among algorithm versions offering sufficient performance, we chose simplicity of use as the criterion to select the final version.

### External Validation of the Triage Algorithm

For each patient from the testing dataset, and irrespective of the presence of priority variables, we calculated the EVD prediction score by summing individual scores of variables present. When the yes/no value of a scoring predictor was missing (20.2% of patients had >1 scoring variable missing), we assigned an individual score of zero (for that variable), assuming the said predictor was likely absent. We excluded patients missing time-to-presentation (589 patients, 4.1%) from this external validation.

We evaluated classification performance by using sensitivity, specificity, positive predictive value (PPV), negative predictive value (NPV), likelihood ratio of positive (LR+) tests, likelihood ratio of negative (LR–) tests, and AUROC to predict an EVD-positive diagnosis by RT-PCR (reference standard). Because of missing data among the 4 priority variables in the testing dataset, we considered 3 strata of patients: prioritized (high-risk) if any of the 4 priority variables were present (“Yes”); not prioritized if all of these 4 variables were absent (“No”); and unknown when none of the 4 variables had “Yes” but >1 was missing.

### Prospective Evaluation

A prospective study evaluated this triage tool in real-life conditions. It was implemented as successive substudies, enabling a target sample size of 65 EVD-positive patients (to estimate sensitivity and specificity with 6% precision) (*23*). We present interim findings from 2 substudies during the 10th (Eastern Democratic Republic of the Congo [DRC], 2020) and 11th (Equateur, DRC, 2020) Ebola epidemics. Our objectives were to evaluate the performance of the developed algorithm in classifying EVD risk among patients matching the Ebola case definition, with GeneXpert RT-PCR as the reference diagnosis, and to assess ease of use by healthcare workers. Participants were patients validated by response teams as matching the Ebola case definition and consenting to be included in the study. Study sites were health facilities where the study was implemented (8 facilities during the 10th DRC epidemic and 9 facilities during the 11th DRC epidemic). 

Before study start, healthcare workers (HCWs) responsible for triage received a 4-hour training session on study ethics, the informed consent process, study procedures (including history-taking and clinical evaluation), investigation of possible exposure histories, the triage algorithm, and study forms. A supervisory staff visited these HCWs a minimum of 3 times following training. HCWs recorded as “present” any sign or symptom occurring at any time since disease onset (even if that symptom had disappeared by the time of evaluation). Healthcare staff recorded exposure histories in the 21 days before disease onset. HCWs evaluated all study participants >1 time, immediately after inclusion, and evaluated some patients again, 24 hours later. The risk categorization did not change the procedures of isolation and testing.

After paper-based data collection, data clerks double entered data in the REDCap electronic data capture tool (https://www.project-redcap.org). We resolved any discrepancies before analyses. We computed concordance between in silico–computed and HCW-computed time-to-presentation, EVD prediction score, and EVD risk category as indicators of quality and comprehension by HCWs. We performed all analyses by using R statistical software (*24*).

## Results

### Diagnostic Performance of Individual Predictors (Appendix Table 1)

In univariate analyses, the predictors offering the best sensitivities were asthenia (80.6%, 95% CI 78.8%–82.4%), anorexia (70.2%, 95% CI 68.0%–72.2%), and being a contact of an EVD case-patient (65.4%, 95% CI 63.1%–67.7%). Taken individually, other predictors, such as fever, had lower sensitivities, approaching or below 50%. Being a contact of an EVD case-patient was the most sensitive predictor for short time-to-presentation (80.2%, 95% CI 76.3%–83.7%), followed by asthenia (70.0%, 95% CI 65.9%–73.9%). Being a contact of an EVD case-patient had a specificity of 83.9% (95% CI 83.4%–84.4%), and other exposure histories had specificities of 90%–99%. In contrast, predictors having the highest sensitivities had lower specificities individually: asthenia at 31.2% (95% CI 30.6%–31.8%) and anorexia at 36.7% (95% CI 36.0%–37.3%).

Because of the low prevalence of EVD in the study population (4%), NPVs were high for all predictors (≥90%) but were the highest for being a contact of an EVD case-patient (96.7%, 95% CI 96.4%–97.0%), having attended a funeral (94.7%, 95% CI 94.4%–95.0%), and having asthenia (94.9%, 95% CI 94.3%–95.4%). Conversely, PPVs were low in general but were highest for bleeding at an injection site (70.6%, 95% CI 56.2%–82.5%), bleeding gums (43.7%, 95% CI 35.8%–51.8%), conjunctivitis (24.6%, 95% CI 21.4%–28.0%), being a contact of an EVD case patient (25.2%, 95% CI 23.9%–26.5%), and having attended a funeral (26.7%, 95% CI 24.9%–28.7%).

Two signs offered high (>8) LR+, bleeding at an injection site (25, 95% CI 13.7–45.5) and bleeding gums (8.1, 95% CI 5.9–11.0), although only for the long time-to-presentation group. Being a contact of an EVD case patient (4.1, 95% CI 3.9–4.3) and having attended a funeral (4.6, 95% CI 4.3–5.0) had lower LR+ (≈4), but those variables were relatively constant regardless of time-to-presentation. We noted negative predictors having LR+ <0.3 (arbitrary threshold) only in the short time-to-presentation group: epistaxis (0.3, 95% CI 0.1–0.8) and melena (0.3, 95% CI 0.1–0.6). Other negative predictors (i.e., abdominal pain, diarrhea for short time-to-presentation, cough, headache) had LR+ >0.5.

### Choice of Predictors

Based on these findings and on those from prior work (*22*), we considered persons at high-risk (prioritization rule) to be those demonstrating any of the following 4 variables: 1) being a contact of an EVD case patient; 2) bleeding at the injection site*;* 3) having bleeding gums*;* and 4) having had contact with an informal healer (outside the health pyramid; eg, a private nurse or traditional healer). This 4th characteristic was not strongly associated with EVD in the database (reporting bias) but strongly linked with superspreading events in investigational reports (data not shown).

To classify patients not demonstrating any of the 4 predictors, we developed an EVD-prediction score based on the 2 described time-to-presentation groups and the remaining predictors having sufficient diagnostic performance and prevalence in >1 of the 2 time-to-presentation periods. Overall, AUROC resulting from application of various sets of predictors and individual scores on bootstrapped samples was 65.3%–73.9% for short time-to-presentation and 70.6%–74.4% for long time-to-presentation (Appendix). Using the new algorithm version 4.2 across 13 variables, we obtained the best compromise between performance and ease of use, with a *+1* individual score assigned for odds ratios (ORs) >1, a *−1* individual score for ORs <1, and a *nil* score for ORs of 1 or ≈1. The resulting AUROC of this scoring component version 4.2 was 71.4% (95% CI 69.8%–72.9%) for short time-to-presentation and 73.3% (95% CI 72.3%–74.4%) for long time-to-presentation (Appendix Tables 2–4).

### Choice of 2 Thresholds

**Figure 1:**
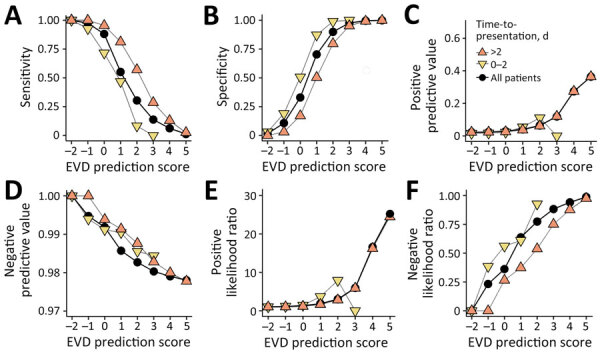
Performance of a rapid decision algorithm for patient triage during Ebola outbreaks (version 4.2, Ebola virus disease [EVD] prediction score only) for different decision thresholds to predict Ebola infection in a population of EVD-suspected patients in Democratic Republic of the Congo during epidemics in 2018–2019, with and without stratification by time-to-presentation (days). A) Sensitivity; B) specificity; C) positive predictive value; D) negative predictive value; E) positive likelihood ratio; F) negative likelihood ratio.

**Figure 2:**
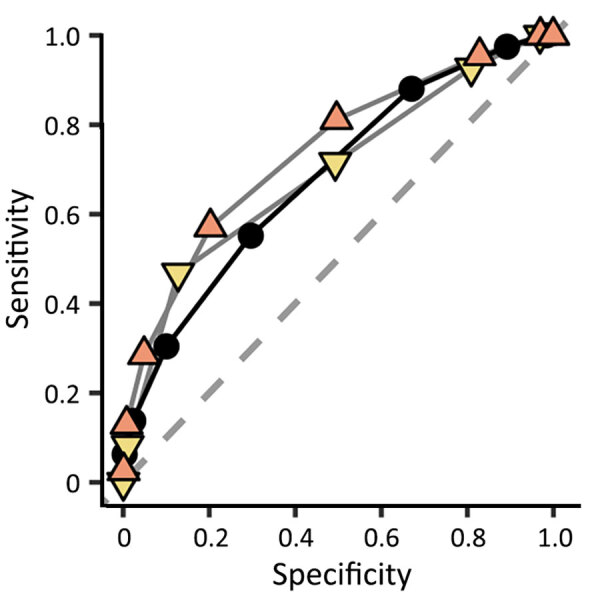
Classification performance (area under the receiver operating characteristic curve) of a rapid decision algorithm for patient triage during Ebola outbreaks a population of EVD-suspected patients in Democratic Republic of the Congo, during epidemics in 2018–2019. Results were based on data from different decision thresholds to predict Ebola infection (Ebola virus disease prediction score only).

We evaluated only performances of the EVD-prediction score (excluding priority variables) on the testing population (Table 1; Figure 1). The AUROC was 68.3% (95% CI 67.9%–68.7%) (Figure 2). Because of the low prevalence of EVD, the NPV was consistently in the range of 98% to 100%, regardless of the value used as a threshold. Regarding negative EVD prediction scores, we anticipated the false-negative rate of <1%, given the situation of low EVD prevalence. However, according to our projections, we surmised that a slight decrease in NPV could also be anticipated in higher-prevalence settings (NPV = 92% for negative EVD-prediction scores in a setting with a confirmation rate of 20% [data not shown]). Conversely, the PPV was low in the lowest values of the prediction score but showed an inflection point from a threshold ≥3 when the PPV reached 10% (Figure 1). For the same threshold, however, the PPV could be expected to rise above 60% if prevalence increased to 20% (data not shown).

**Table 1:** Diagnostic performance statistics when applying the EVD-prediction score of a rapid decision algorithm for patient triage during Ebola outbreaks to a testing population derived from EVD-suspected patients, Democratic Republic of the Congo, during epidemics in 2018–2019*

Parameter	Threshold							
	≥−2	≥−1	≥0	≥1	≥2	≥3	≥4	≥5
Sensitivity, % (95% CI)	100 (98.8–100)	97.5 (95.1–98.9)	88.1 (84.0–91.4)	55.1 (49.5–60.7)	30.4 (25.4–35.8)	13.8 (10.2–18.1)	6.3 (3.9–9.5)	1.3 (0.3–3.29)
Specificity, % (95% CI)	1.6 (1.4–1.7)	10.8 (10.3–11.3)	33.0 (32.2–33.7)	70.4 (69.6–71.1)	89.9 (89.4–90.4)	97.7 (97.4–97.9)	99.6 (99.5–99.7)	100 (99.9–100)
PPV, % (95% CI)	2.3 (2.0–2.5)	2.4 (2.2–2.7)	2.9 (2.6–3.3)	4.1 (3.5–4.7)	6.4 (5.2–7.8)	11.9 (8.8–15.7)	27.4 (17.6–39.1)	36.4 (10.9–69.2)
NPV, % (95% CI)	100 (98.3–100)	99.5 (99.0–99.8)	99.2 (98.9–99.4)	98.6 (98.3–98.8)	98.3 (98.0–98.5)	98.0 (97.8–98.3)	97.9 (97.7–98.1)	97.8 (97.5–98.0)
LR+ (95% CI)	1.0 (1.0–1.0)	1.1 (1.1–1.1)	1.3 (1.3–1.4)	1.9 (1.7–2.1)	3.0 (2.5–3.6)	6.0 (4.4–8.0)	16.6 (10.0–27.4)	25.1 (7.4–85.4)
LR− (95% CI)	0.0 (0.0–0.0)	0.23 (0.12–0.46)	0.36 (0.27–0.49)	0.64 (0.56–0.72)	0.77 (0.72–0.83)	0.88 (0.84–0.92)	0.94 (0.91–0.97)	0.99 (0.98–1.0)

*The algorithm (version 4.2) considered all times-to-presentation (time from symptom onset to seeking care). EVD, Ebola virus disease; LR−, likelihood ratio of negative; LR+, likelihood ratio of positive; NPV, negative predictive value; PPV, positive predictive value.

Likelihood ratios represent the multiplicative factor converting the pretest probability into a posttest probability of infection. They do not vary with prevalence and can be used at the individual level. Three thresholds (≥3, ≥4, ≥5) provided sufficient LR+, and 2 other thresholds (≤−2, ≤−1) provided adequate LR– (Table 1).

Of note, when inspecting separately the performances for the 2 time-to-presentation groups, sensitivity was slightly higher for long time-to-presentation than for short time-to-presentation. This finding would lead to lower performance detecting EVD-positive cases shortly after disease onset. Both LR+ and LR– also performed better for long time-to-presentation.

**Figure 3:**
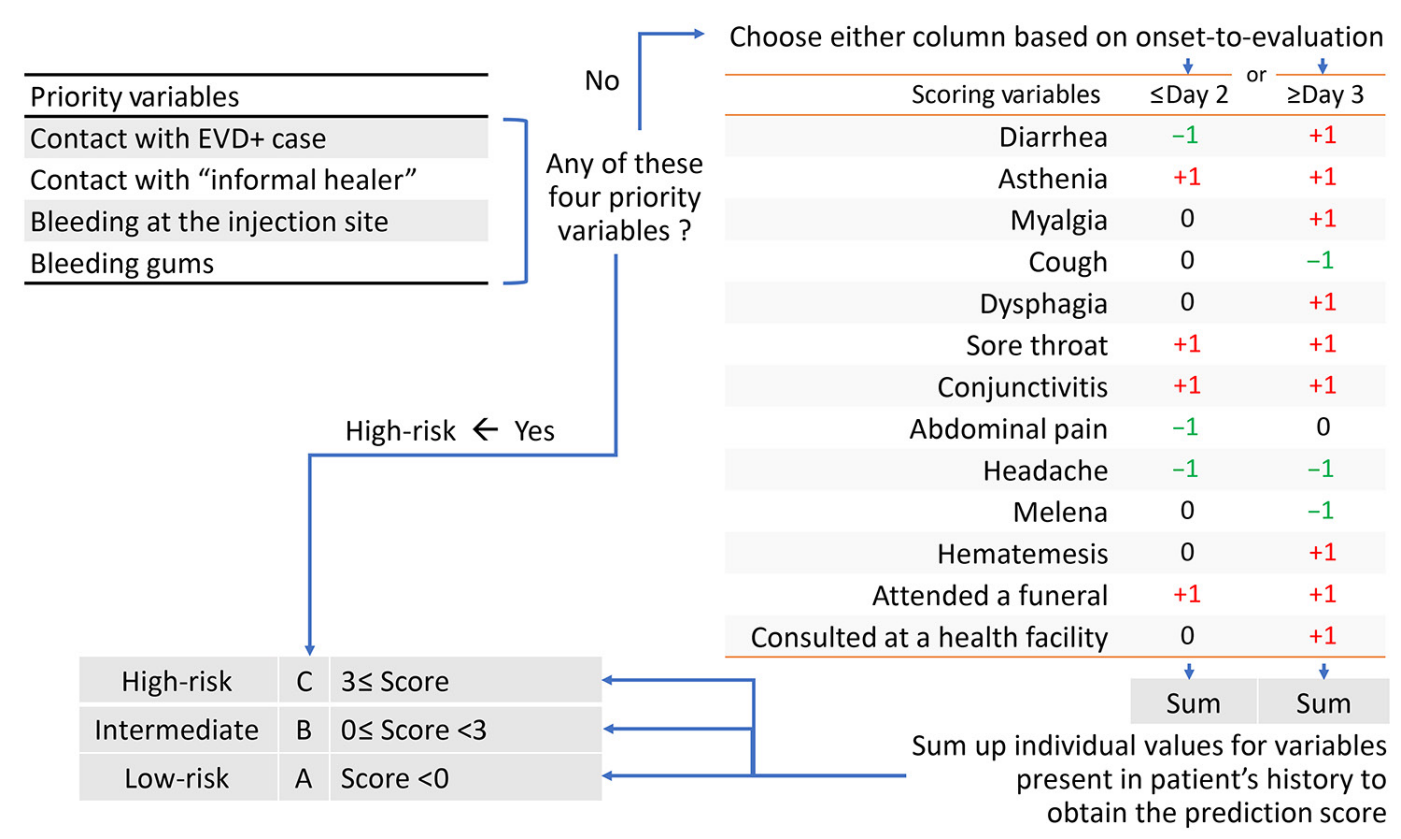
Final triage algorithm (version 4.2) for the evaluation of the likelihood of Ebola infection among EVD-suspected patients in Democratic Republic of the Congo, during epidemics in 2018–2019. Three risk categories of Ebola infection are defined: A category (low-risk), B category (intermediate-risk), or C category (high-risk). Left: 4 priority variables. Right: 2 sets of individual scores for each of the 13 variables, to be chosen according to time-to-presentation (thresholds at the top of columns). For methods of employing algorithm, see Appendix. EVD, Ebola virus disease; EVD+, EVD-positive.

Given those expected performances and numbers of patients in each category, we determined the most suitable thresholds (0; 2) to be for the intermediate-risk category (Figure 3). For the lowest threshold, we favored the combination of an optimal size of risk categories with the highest sensitivity to reduce the false-negative rate.

### Positivity Rates and Viral Loads

**Figure 4:**
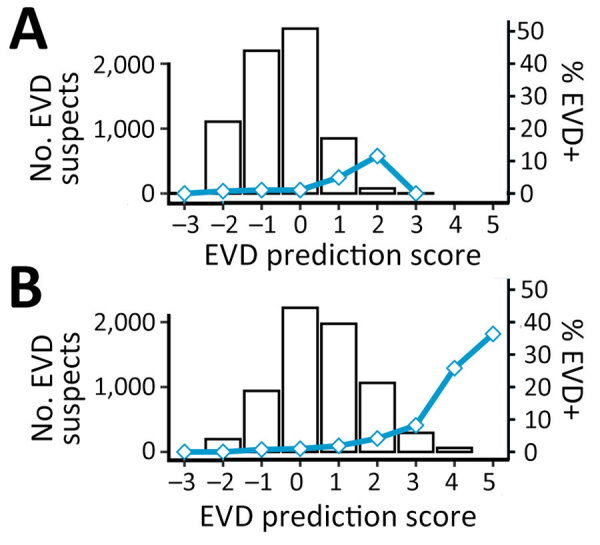
EVD confirmation rates (blue line) and number of patients (bars) classified by EVD prediction score obtained by a rapid decision algorithm for patient triage during Ebola outbreaks used in a population of EVD-suspected patients in Democratic Republic of the Congo during epidemics in 2018–2019. EVD, Ebola virus disease; EVD+, EVD-positive.

**Figure 5:**
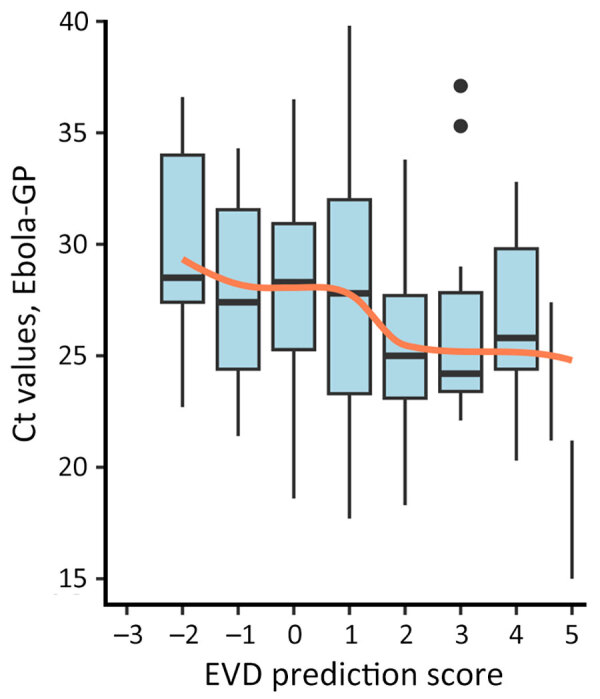
Distribution of Ebola GP cycle threshold Ct values among EVD-positive patients and averages of Ct values (orange line) by EVD prediction score obtained by a rapid decision algorithm for patient triage during Ebola outbreaks used in a population of EVD-suspected patients in Democratic Republic of the Congo during epidemics in 2018–2019. Box plots indicate medians (horizontal black lines), interquartile range (box tops and bottoms), and 95% CIs (error bars); black dots indicate outliers. Ct, cycle threshold; EVD, Ebola virus disease; GP, glycoprotein.

When applying the scoring component of our new version 4.2 algorithm to the testing population, EVD prediction scores were distributed around a mode of zero, and we observed a slight shift to higher values for long time-to-presentation. As expected, EVD confirmation rates were associated with EVD prediction scores (Table 2, Figure 4) and their distribution confirmed the choice of the 2 thresholds. From the score +1, this rate reached or exceeded the average in all 3 subgroups: prioritized, unknown (missing data for some priority variables), and nonprioritized patients. A clear trend of increasing viral load with increasing EVD prediction scores (Figure 5) suggested that these parameters reflected not only the likelihood of infection but also disease severity.

**Table 2:** EVD confirmation rates and total number of patients classified by a rapid decision algorithm for patient triage during Ebola outbreaks and time-to-presentation based on a population of EVD-suspected patients, Democratic Republic of the Congo, during epidemics in 2018–2019*

Stratification of testing population	Prediction scores, % (no.)									
	All scores	−3	−2	−1	0	1	2	3	4	5
Short time-to-presentation										
No stratification	1.6 (6,993)	0.0 (215)	0.7 (1,107)	1.1 (2,200)	1.1 (2,541)	5.0 (849)	11.5 (78)	0.0 (3)		
Prioritized	6.8. (740)	0.0 (10)	4.0 (76)	2.6 (195)	3.8 (261)	14.7 (163)	24.2 (33)	0.0 (2)		
Not prioritized	0.2 (4,471)	0.0 (159)	0.0 (759)	0.1 (1,496)	0.1 (1,504)	0.6 (522)	0.0 (30)	0.0 (1)		
Unknown	2.9 (1,782)	0.0 (46)	1.8 (272)	3.1 (509)	1.9 (776)	9.2 (164)	6.7 (15)	NA		
Long time-to-presentation										
No stratification	2.3 (6,765)	0.0 (3)	0.0 (197)	0.7 (940)	1.0 (2,221)	1.9 (1,975)	4.1 (1,063)	8.2 (293)	25.8 (62)	36.4 (11)
Prioritized	17.3 (468)	NA	0.0 (9)	4.0 (50)	7.32 (123)	13.4 (112)	24.0 (100)	37.5 (48)	52.4 (21)	40.0 (5)
Not prioritized	0.8 (4,629)	0.0 (2)	0.0 (157)	0.4 (702)	0.5 (1,468)	0.7 (1,377)	1.4 (706)	1.1 (185)	10.7 (28)	25.0 (4)
Unknown	2.2 (1,668)	0.0 (1)	0.0 (31)	1.1 (188)	0.8 (630)	2.7 (486)	3.9 (257)	6.7 (60)	15.4 (13)	50.0 (2)

*The algorithm (version 4.2) considered all times-to-presentation **(**time from symptom onset to seeking care**).** The testing population is either not stratified or stratified into those prioritized, those not prioritized, or unknown (see Methods). NA, not applicable.

### Performance of Priority Variables

In the testing population, 1,302 patients (9.1%) had >1 of the 4 priority variables, 9,411 (65.6%) had none, and 3,633 (25.3%) had none but with some missing information. We looked at the prevalence of each of the 4 characteristics among EVD-positive and -negative patients, in particular the much higher frequency of these characteristics among the EVD-positive persons (Appendix Table 5). Adding this prioritization rule, we then multiplied the size of the high-risk category by about 4 to 8 (compared with the EVD prediction score only), depending on the age group considered (Table 3), with relatively steady confirmation rates. We noted, however, that sensitivities of the lower and upper thresholds in all age groups were significantly improved: lower threshold was 88.1% (95% CI 84.0%–91.4%) and upper threshold 13.8% (95% CI 10.2%–18.1%) without the prioritization rule; and lower threshold 91.2% (95% CI 88.1%–94.3%) and upper threshold 56.7% (95% CI 51.3%–62.2%) with the prioritization rule. Of note, NPV of the low-risk category was highest (100%) in children 0–5 years of age. Of the testing population, the low-risk category represented 30.1% (95% CI 29.4%–30.9%), the intermediate-risk category represented 58.8% (95% CI 57.9%–59.6%), and the high-risk category represented 11.1% (95% CI 10.6%–11.6%).

**Table 3:** EVD-positive rates and number of EVD-positive and EVD-suspected patients by category of infection after applying a rapid decision algorithm for patient triage during Ebola outbreaks on a testing dataset based on a population of EVD-suspected patients in Democratic Republic of the Congo during epidemics in 2018–2019*

Age category, y	EVD+ rate, % (no.)	Algorithm version 4.2 components	EVD+ rates by EVD-risk classification, % (n/N)		
			Low (A)	Intermediate (B)	High (C)
All ages	2.2 (319/14,346)	Scoring only	0.82 (38/4,462)	2.54 (237/9,315)	11.92 (44/369)
		Scoring + priority variables	0.65 (28/4,322)	1.31 (110/8,429)	11.35 (181/1,595)
0–5	1.6 (43/2,618)	Scoring only	0 (0/710)	2.13 (40/1,878)	10.0 (3/30)
		Scoring + priority variables	0 (0/671)	1.56 (27/1,730)	7.37 (16/217)
6–12	1.2 (24/1,960)	Scoring only	1.02 (8/784)	1.22 (14/1,152)	8.33 (2/24)
		Scoring + priority variables	0.41 (3/724)	0.66 (7/1,066)	8.24 (14/170)
13–25	1.5 (64/4,366)	Scoring only	0.55 (9/1,650)	1.69 (44/2,600)	9.48 (11/116)
		Scoring + priority variables	0.33 (5/1,538)	0.88 (21/2,385)	8.58 (38/443)
>25	3.5 (187/5,386)	Scoring only	1.38 (21/1,518)	3.76 (138/3,669)	14.07 (28/199)
		Scoring + priority variables	1.44 (20/1,389)	1.67 (54/3,232)	14.77 (113/765)

*EVD, Ebola virus disease; EVD+, EVD-positive.

### Comparison with Other Classifiers

We compared performances of our newly developed algorithm (version 4.2) with those from previous work (*15*,*17*,*19*) based on our testing population. Because of the prioritization rule, the sensitivity of our algorithm could not drop below 50% in lowest scores and, conversely, its specificity was limited to 90% in highest scores. This limitation prevented a comparison of our AUROC results with those of other tools. All algorithms showed PPV increasing with prediction scores except Oza’s version (Appendix Figure 1). In their highest prediction scores, Hartley and Levine’s versions seemed to have suitable PPV and LR+ for late presenters but not for early presenters. Compared with other developed tools, our new algorithm also seemed to show more favorable results in terms of NPV and LR–.

### Interim Results of the Prospective Study

We investigated data obtained from 2,652 EVD-suspected patients involved in the 10th (N = 2,206) and 11th (N = 446) DRC Ebola epidemics. We also compiled information in 2 substudies, where 102 patients underwent a second evaluation after 24 hours. After excluding patients with missing data, we focused our analysis on a total of 2,695 evaluations (Appendix Table 6, Figure 2).

The triage tool procedure appeared understandable to health personnel; however, some aspects of the history-taking process required further instruction. Because HCWs often were not familiar with ongoing chains of transmission, nor with step-by-step approaches for such investigations, supervision appeared crucial to improve the collection of variables, especially exposure histories to potential Ebola case-patients. Tool assessment showed good concordance; 95.8% of evaluation forms had correct calculation of time-to-presentation, 98.5% had a correct choice of the set of individual predictors, and 95.3% showed EVD scores correctly computed. Quality of patient evaluation at triage required good engagement between health personnel and the supervisory study team.

Eight EVD-positive patients were identified in the study population, 2 of whom had received Ebola vaccination (Table 4). EVD prediction scores were above +2 in 2 of the 8 cases, and detection of some of the 4 priority variables reclassified 3 of the 8 cases into the high-risk category. We therefore classified 5 of the 8 cases as high-risk and the other 3 as intermediate-risk. According to the collected variables, the EVD prediction scores and risk categories calculated by HCWs were correct; however, a review of narratives from investigation teams revealed difficulties in detecting some exposure histories.

**Table 4:** Summary of EVD-positive cases included in the prospective study of a rapid decision algorithm for patient triage during Ebola outbreaks using EVD-suspected patients in Democratic Republic of the Congo during epidemics in 2018–2019*

Case no.	Age, mo/sex	Time-to-presentation, d	Vaccinated	Priority variables	Scoring variables	Prediction score			Risk classification	
						Staff	In silico		Staff	In silico
1	15/F	3	No	Contact EVD case	Asthenia, headache	0	0		C	C
2	47/F	5	Yes	Contact EVD case	Attended a funeral, asthenia, myalgia, dysphagia, sore throat	5	5		C	C
3	39/F	1	Yes	Contact EVD case	Headache	−1	−1		C	C
4	27/F	3	No	NA	Asthenia, headache, abdominal pain, diarrhea	1	1		B	B
5	24/F	6	No	NA	Diarrhea	1	1		B	B
6	33/F	10	No	Contact EVD case, contact informal healer	Asthenia, dysphagia, sore throat, diarrhea	4	4		C	C
7	78/M	8	No	NA	Attended a funeral, myalgia, abdominal pain, diarrhea, melena	2	2		B	B
8	9/F	8	No	Contact EVD case	Asthenia (coma), headache, abdominal pain	0	0		C	C

*Three risk categories of Ebola infection are defined: A category (low-risk), B category (intermediate-risk), or C category (high-risk). Age, sex, time-to-presentation, vaccination status, collected exposure histories, clinical signs, and symptoms (data collected by health personnel at the triage of health facilities) and risk-categorization (by healthcare personnel or in silico) are displayed. EVD, Ebola virus disease; NA, not applicable.

Considering all included EVD-suspected patients, and as also seen with the retrospective data, we observed an increase in the likelihood of infection by EVD risk category (Table 5), and the relative sizes of the 3 risk categories were similar to those obtained with the testing dataset. Of note, at this interim stage, the low-risk category (38.9% of evaluated patients, 95% CI 37.1%–40.8%) included no EVD-positive cases.

**Table 5:** EVD confirmation rates and number of all EVD+ and EVD− patients included in a rapid decision algorithm for patient triage during Ebola outbreaks in Democratic Republic of the Congo, during epidemics in 2018–2019*

EVD status	Risk classification			Total
	Low	Intermediate	High	
EVD−, noncase, no. cases	1,050	1,374	263	2,687
EVD+, PCR-confirmed, no. cases	0	3	5	8
Total, no. (%) cases	1,050 (38.9)	1377 (51.1)	268 (9.9)	2,695 (100)
EVD confirmation rate	0.0%	0.2%	1.9%	0.3%

*EVD, Ebola virus disease; EVD+, EVD-positive; EVD−, EVD-negative.

## Discussion

We developed a decision algorithm that classified Ebola risk into 3 categories to enable more rapid identification of patients most at risk for EVD during outbreaks. We favored sensitivity of the lower threshold to avoid false negatives in the low-risk category. This tool combined 4 priority variables, 2 time-to-presentation periods from symptom onset, and the predictors most strongly associated with EVD. Although the predictors had insufficient performance individually, their combination into a scoring tool enabled the classification of patients by infection likelihood, using both retrospective and prospective data. The EVD prediction score was also positively correlated with viral load, reflecting disease severity. The examination of score performance established 2 optimal thresholds differentiating 3 risk categories. Those parameters yielded relatively similar performance for early and late presentation, although results were slightly better for long time-to-presentation. Using retrospective data, we observed that the low-risk category comprised 30.1% and the high-risk category 11.1% of all EVD-suspected patients. Applying 4 priority variables to the data drove a significant increase in sensitivity of the high-risk category (56.7% with those variables vs. 13.8% without) without decreasing the PPV. In parallel, the addition of those variables increased the sensitivity of the lower threshold from 88.1% to 91.2%, reducing the false-negative rate. Our prospective study broadly confirmed these findings, with a similar distribution of patients in the risk categories and an increase in EVD confirmation rates across risk groups. At this interim stage of our investigation, no EVD-positive case was classified as low-risk by the algorithm.

With efficient treatments now available, and given that EVD proceeds rapidly toward irremediable sequelae or death, prioritizing higher-risk patients for RT-PCR testing (high-risk, then intermediate-risk) would likely improve Ebola outcomes (*1*,*14*). In addition, downstream contacts of case-patients could be followed up sooner. Quickly diagnosing high-risk patients could benefit not only case management but also the efficiency of the outbreak response, as shown by modeling the use of rapid diagnostic tests for early triage (*25*). Synergistic effects could be expected with the recent improvements in vaccination (*26*), decentralized care, or specific treatments (*27*).

We also showed that it is feasible to identify a significant proportion of persons at low risk for Ebola infection. This group could benefit from lighter isolation measures, with daily clinical re-evaluation, allowing for better differential diagnosis and appropriate care. Nevertheless, adequate prevention measures should be maintained until final status is established through laboratory testing or exclusion on clinical grounds.

Identifying exposures to a known EVD-positive case-patient considerably increases performance of infection prediction. Our study team’s close work with triage personnel revealed the difficulty of properly assessing such risk factors. In practice, investigation and response teams are well aware of Ebola transmission chains, but at the health facility level, HCWs often do not know about them. In addition, patients can only share details of their potential exposures if they both understand EVD transmission routes and trust the rationale of control measures. Based on those assumptions, reducing nosocomial transmission in regular (non-Ebola) health facilities might be fostered by training health staff on leading discussions and investigating potential exposures to possible Ebola cases before outbreaks and communicating transmission chains from the previous 3 weeks during outbreaks.

One limitation of our study is that our algorithm (version 4.2) has not yet been implemented prospectively where Ebola incidence is high. According to our estimates, NPV of the low-risk category could decrease to 92% for a prevalence of 20% among EVD-suspected patients. In addition, the comprehensive interpretation of the performance of this triage algorithm, when applied prospectively, requires that the target sample size be reached.

In conclusion, the current case definition and subdefinitions for Ebola suspicion are broad, requiring that any febrile patient having 3 signs or symptoms be isolated for >48 hours and tested 2 times to rule out EVD before further biomedical investigations can be performed. Previous evidence has demonstrated lack of sensitivity and mainly specificity for this approach (*6*,*28*–*30*). Our results suggest that, with minimal training of investigational personnel, simple clinical and epidemiologic criteria can reliably establish the probability of Ebola infection among EVD-suspected patients. Therefore, we propose that EVD-suspected patients be considered by risk for infection, rather than in an undifferentiated manner. At the level of standard health facilities, such a tool and knowledge would support confident decision-making by health personnel and likely reduce nosocomial transmission. In addition, simple rules—such as prioritization of RT-PCR for the most at-risk patients or lighter isolation measures and differential diagnosis investigation for the least at-risk patients—could improve quality of care and favor outbreak control. Finally, by adapting prevention and testing measures by likelihood of infection, community acceptance and participation could be greatly improved.

Appendix More information for rapid decision algorithm for patient triage during Ebola outbreaks, including the method for employing the version 4.2 triage algorithm.
