# Supplementary material for: Rapid Decision Algorithm for Patient Triage during Ebola Outbreaks
Source: Emerg Infect Dis. 2024 Nov;30(11):e231650. doi: 10.3201/eid3011.231650 (PMC11521189; doi:10.3201/eid3011.231650)
Supplement: Appendix — More information for rapid decision algorithm for patient triage during Ebola outbreaks, including the method for employing the version 4.2 triage algorithm. [file 23-1650-Techapp-s1.pdf]

Article DOI: http://doi.org/10.3201/eid3010.231650

EID cannot ensure accessibility for supplementary materials supplied by authors. Readers who have difficulty accessing supplementary content should contact the authors for assistance.

Rapid Decision Algorithm for Patient Triage during Ebola Outbreaks

Appendix

Method for Employing the Algorithm

The method for using the algorithm (Figure 3) was as follows: a patient with any of the four “priority” variables was considered as high-risk of infection (C category) regardless of his/her score (“prioritization rule”). The EVD-prediction score (right-side of the tool) was computed by summing up the individual scores of each sign, symptom, or exposure history noted as “present”, after which the A/B/C categorization followed the thresholds indicated on the bottom-left; the rational choice of these thresholds is explained in the result section.

Characteristics of the Testing Population

The testing population consisted of 14,346 new EVD-suspected patients, including 2,618 children under 5 (18.3%), notified between August 15, 2019, and November 27, 2019, of whom 319 were confirmed EVD-positive. The confirmation rate was therefore 2.2%, around 3.6 times lower than that observed in the testing dataset. As with the modeling population, there was a higher positivity rate among late presenters than among early presenters (109/6993 = 1.6% if notified within days 0-2 of symptoms, versus 154/6765, 2.3% if notified at day 3 after onset or later).

In this testing population, 1140/11500 patients (9.9%) had had contact with a known EVD case, among whom the RT-PCR confirmation rate was 13.4%. In the other subjects, the confirmation rate was only 0.6%. The confirmation rate in the presence of the other three priority variables was also higher than in their absence (contact with a traditional practitioner: 7.1% vs. 1.7%; bleeding at the injection site: 17.6% vs. 2.2%; bleeding from the gums: 24.4% vs. 2.2%).

Finally, logistic regression showed a profile broadly similar to that obtained in the modeling phase (*22*): among patients notified within the first two days following symptom onset, contact with a known EVD case (OR = 4.0, IC95%: 1.7-10.1), recent attendance at a funeral (OR = 2.6, IC95% = 1.4-5.0) , contact with a health care facility (OR = 2.3, IC95%: 0.9-5.1), bleeding gums (OR = 23.8, IC95% = 0. 8-317), and asthenia (OR = 2.9, IC95% = 1.5-6.0) were positive predictors of infection, while abdominal pain (OR = 0.3, IC95% = 01-0.6) was a negative predictor. Among patients notified 3 days or more after the onset of symptoms, contact with an EVD case (OR = 27.2, IC95% : 16.3-45.9), a health facility (OR = 4.6, IC95% : 2.7-7.5) and a traditional practitioner (OR = 3.4, IC95% : 1.0-9.6) positively predicted infection, as did bleeding at the injection site (OR = 144, IC95% : 19. 3-1238), conjunctivitis (OR = 8.2, IC95% : 3.5-18.4), nausea/vomiting (OR = 1.6, IC95% : 1.0-2.6), vomiting of blood (OR = 3.4, IC95% : 0.9-10.0), asthenia (OR = 2. 8, IC95%: 1.5-5.6), anorexia (OR = 2.1, IC95%: 1.2-3.8), throat pain (OR = 2.6, IC95%: 1.2-5.4) and muscle/joint pain (OR = 1.7, IC95%: 1.0-3.0). Conversely, cough (OR = 0.6, IC95%: 0.4-1.0) and headache (OR = 0.6, IC95%: 0.4-1.0) were negative predictors in late presenters.

Appendix Table 1. Diagnostic performance of individual variables for the prediction of Ebola infection (Training dataset)

Variable	Time-to-presentation	**Sensitivity** (95%CI)			**Specificity** (95%CI)			**PPV** (95%CI)			**NPV** (95%CI)			**LR+** (95%CI)			**LR-** (95%CI)		
Abdominal pain	Any	**46.7%**	44.4%	49.0%	**43.7%**	43.1%	44.4%	**6.8%**	6.3%	7.2%	**90.4%**	89.8%	90.9%	**0.8**	0.8	0.9	**1.2**	1.2	1.3
Anorexia	Any	**70.2%**	68.0%	72.2%	**36.7%**	36.0%	37.3%	**8.8%**	8.4%	9.3%	**93.4%**	92.8%	93.9%	**1.1**	1.1	1.1	**0.8**	0.8	0.9
Asthenia	Any	**80.6%**	78.8%	82.4%	**31.2%**	30.6%	31.8%	**9.2%**	8.8%	9.7%	**94.9%**	94.3%	95.4%	**1.2**	1.1	1.2	**0.6**	0.6	0.7
Any bleeding	Any	**14.5%**	12.9%	16.2%	**86.1%**	85.7%	86.6%	**8.3%**	7.3%	9.3%	**92.1%**	91.7%	92.5%	**1.0**	0.9	1.2	**1.0**	1.0	1.0
Bleeding epistaxis	Any	**3.0%**	2.3%	3.9%	**96.1%**	95.8%	96.3%	**6.9%**	5.2%	8.9%	**91.2%**	90.8%	91.6%	**0.8**	0.6	1.0	**1.0**	1.0	1.0
Bleeding gums	Any	**3.9%**	3.0%	4.9%	**99.5%**	99.4%	99.6%	**43.7%**	35.8%	51.8%	**91.5%**	91.1%	91.9%	**8.1**	5.9	11.0	**1.0**	1.0	1.0
Bleeding hemoptisis	Any	**0.9%**	0.5%	1.5%	**98.9%**	98.8%	99.1%	**7.5%**	4.3%	11.9%	**91.2%**	90.8%	91.6%	**0.8**	0.5	1.4	**1.0**	1.0	1.0
Bleeding injection site	Any	**2.0%**	1.4%	2.8%	**99.9%**	99.9%	100.0%	**70.6%**	56.2%	82.5%	**91.4%**	91.0%	91.8%	**25.0**	13.7	45.5	**1.0**	1.0	1.0
Bleeding melena	Any	**4.0%**	3.2%	5.1%	**93.9%**	93.5%	94.2%	**5.9%**	4.6%	7.4%	**91.1%**	90.7%	91.5%	**0.7**	0.5	0.8	**1.0**	1.0	1.0
Bleeding other	Any	**1.8%**	1.3%	2.6%	**98.8%**	98.7%	99.0%	**12.6%**	8.8%	17.2%	**91.6%**	91.2%	92.0%	**1.6**	1.1	2.2	**1.0**	1.0	1.0
Bleeding urine	Any	**0.6%**	0.3%	1.0%	**99.1%**	98.9%	99.2%	**5.4%**	2.6%	9.7%	**91.2%**	90.8%	91.6%	**0.6**	0.3	1.1	**1.0**	1.0	1.0
Bleeding vagina	Any	**2.2%**	1.6%	3.0%	**96.2%**	95.9%	96.5%	**5.4%**	3.9%	7.2%	**91.1%**	90.7%	91.5%	**0.6**	0.4	0.8	**1.0**	1.0	1.0
Bleeding vomito negro	Any	**0.8%**	0.5%	1.4%	**99.5%**	99.4%	99.6%	**13.9%**	8.0%	21.9%	**91.2%**	90.8%	91.6%	**1.7**	1.0	2.9	**1.0**	1.0	1.0
Bleeding hematemesis	Any	**3.5%**	2.7%	4.5%	**97.6%**	97.4%	97.8%	**12.4%**	9.6%	15.6%	**91.3%**	90.9%	91.7%	**1.5**	1.1	1.9	**1.0**	1.0	1.0
Breathlessness	Any	**10.8%**	9.4%	12.3%	**90.0%**	89.6%	90.5%	**9.0%**	7.9%	10.3%	**91.7%**	91.3%	92.1%	**1.1**	0.9	1.2	**1.0**	1.0	1.0
Chest pain	Any	**21.5%**	19.7%	23.5%	**84.4%**	83.8%	84.8%	**11.1%**	10.1%	12.2%	**92.2%**	91.8%	92.6%	**1.4**	1.3	1.5	**0.9**	0.9	1.0
Coma	Any	**2.0%**	1.4%	2.8%	**98.3%**	98.1%	98.4%	**9.6%**	6.9%	13.0%	**91.6%**	91.3%	92.0%	**1.2**	0.8	1.6	**1.0**	1.0	1.0
Confusion, disorientation	Any	**3.1%**	2.3%	4.0%	**98.6%**	98.4%	98.7%	**16.5%**	12.7%	20.8%	**91.8%**	91.4%	92.1%	**2.2**	1.6	2.9	**1.0**	1.0	1.0
Conjunctivitis	Any	**9.2%**	7.9%	10.6%	**97.4%**	97.2%	97.6%	**24.6%**	21.4%	28.0%	**92.1%**	91.8%	92.5%	**3.6**	3.0	4.2	**0.9**	0.9	0.9
Cough	Any	**18.8%**	17.0%	20.7%	**69.3%**	68.6%	69.9%	**5.1%**	4.6%	5.7%	**90.6%**	90.1%	91.1%	**0.6**	0.6	0.7	**1.2**	1.1	1.2
Contact EVD case	Any	**65.4%**	63.1%	67.7%	**83.9%**	83.4%	84.4%	**25.2%**	23.9%	26.5%	**96.7%**	96.4%	97.0%	**4.1**	3.9	4.3	**0.4**	0.4	0.4
Contact funeral	Any	**34.4%**	32.1%	36.7%	**92.6%**	92.2%	92.9%	**26.7%**	24.9%	28.7%	**94.7%**	94.4%	95.0%	**4.6**	4.3	5.0	**0.7**	0.7	0.7
Contact health facility	Any	**27.3%**	25.1%	29.5%	**90.1%**	89.7%	90.5%	**17.9%**	16.4%	19.4%	**94.0%**	93.7%	94.3%	**2.8**	2.5	3.0	**0.8**	0.8	0.8
Contact informal healer	Any	**3.2%**	2.4%	4.2%	**99.0%**	98.8%	99.1%	**19.3%**	14.8%	24.6%	**92.9%**	92.6%	93.2%	**3.1**	2.3	4.1	**1.0**	1.0	1.0
Diarrhea	Any	**40.4%**	38.2%	42.7%	**62.9%**	62.2%	63.5%	**8.9%**	8.3%	9.5%	**92.2%**	91.8%	92.6%	**1.1**	1.0	1.2	**0.9**	0.9	1.0
Eyes: light sensitvity	Any	**2.2%**	1.6%	3.0%	**98.5%**	98.3%	98.6%	**11.5%**	8.4%	15.4%	**91.7%**	91.3%	92.1%	**1.4**	1.0	2.0	**1.0**	1.0	1.0
Fever	Any	**49.6%**	47.3%	51.9%	**48.8%**	48.1%	49.4%	**7.7%**	7.2%	8.2%	**91.8%**	91.3%	92.3%	**1.0**	0.9	1.0	**1.0**	1.0	1.1
Headache	Any	**53.1%**	50.8%	55.4%	**38.3%**	37.6%	38.9%	**7.0%**	6.6%	7.4%	**90.4%**	89.7%	91.0%	**0.9**	0.8	0.9	**1.2**	1.2	1.3
Hiccups	Any	**4.3%**	3.4%	5.4%	**97.4%**	97.1%	97.6%	**13.0%**	10.4%	15.9%	**91.8%**	91.4%	92.1%	**1.6**	1.3	2.1	**1.0**	1.0	1.0
Jaundice	Any	**3.5%**	2.7%	4.4%	**97.4%**	97.1%	97.6%	**10.8%**	8.4%	13.6%	**91.7%**	91.3%	92.1%	**1.3**	1.0	1.7	**1.0**	1.0	1.0
Joint pain	Any	**45.1%**	42.8%	47.4%	**63.7%**	63.0%	64.4%	**10.0%**	9.3%	10.6%	**92.9%**	92.4%	93.3%	**1.2**	1.2	1.3	**0.9**	0.8	0.9
Myalgia	Any	**38.1%**	35.9%	40.4%	**71.7%**	71.1%	72.4%	**10.8%**	10.0%	11.5%	**92.8%**	92.4%	93.2%	**1.3**	1.3	1.4	**0.9**	0.8	0.9
Other symptoms	Any	**9.6%**	8.1%	11.4%	**89.9%**	89.4%	90.4%	**7.8%**	6.5%	9.2%	**91.8%**	91.4%	92.3%	**0.9**	0.8	1.1	**1.0**	1.0	1.0
Petechia purpura	Any	**0.4%**	0.2%	0.8%	**99.8%**	99.8%	99.9%	**19.4%**	8.2%	36.0%	**91.3%**	90.9%	91.6%	**2.5**	1.1	5.7	**1.0**	1.0	1.0
Rash	Any	**4.0%**	3.2%	5.1%	**96.8%**	96.6%	97.1%	**10.5%**	8.3%	12.9%	**91.7%**	91.3%	92.1%	**1.3**	1.0	1.6	**1.0**	1.0	1.0
Dysphagia	Any	**18.6%**	16.9%	20.5%	**90.7%**	90.3%	91.1%	**15.4%**	13.9%	17.0%	**92.4%**	92.1%	92.8%	**2.0**	1.8	2.2	**0.9**	0.9	0.9
Sore throat	Any	**15.7%**	14.1%	17.4%	**92.9%**	92.6%	93.3%	**16.8%**	15.1%	18.7%	**92.4%**	92.0%	92.8%	**2.2**	2.0	2.5	**0.9**	0.9	0.9
Vomiting. nausea	Any	**50.6%**	48.3%	52.9%	**49.5%**	48.8%	50.1%	**8.2%**	7.7%	8.7%	**91.9%**	91.3%	92.4%	**1.0**	1.0	1.0	**1.0**	1.0	1.0
Abdominal pain	Days 0-2	**27.6%**	23.8%	31.7%	**45.2%**	44.3%	46.2%	**2.6%**	2.2%	3.0%	**92.3%**	91.5%	93.1%	**0.5**	0.4	0.6	**1.6**	1.5	1.7
Anorexia	Days 0-2	**53.8%**	49.4%	58.1%	**41.0%**	40.1%	42.0%	**4.6%**	4.1%	5.1%	**94.4%**	93.7%	95.1%	**0.9**	0.8	1.0	**1.1**	1.0	1.2
Asthenia	Days 0-2	**70.0%**	65.9%	73.9%	**35.8%**	34.9%	36.7%	**5.4%**	4.8%	5.9%	**95.8%**	95.1%	96.4%	**1.1**	1.0	1.2	**0.8**	0.7	1.0
Any bleeding	Days 0-2	**6.9%**	4.9%	9.5%	**87.4%**	86.7%	88.0%	**2.8%**	2.0%	3.8%	**94.8%**	94.3%	95.2%	**0.6**	0.4	0.8	**1.1**	1.0	1.1
Bleeding epistaxis	Days 0-2	**1.0%**	0.3%	2.3%	**96.3%**	95.9%	96.7%	**1.5%**	0.5%	3.5%	**94.4%**	93.9%	94.9%	**0.3**	0.1	0.6	**1.0**	1.0	1.0

Variable	Time-to-presentation	**Sensitivity** (95%CI)			**Specificity** (95%CI)			**PPV** (95%CI)			**NPV** (95%CI)			**LR+** (95%CI)			**LR-** (95%CI)		
Bleeding gums	Days 0-2	**0.8%**	0.2%	2.0%	**99.6%**	99.5%	99.7%	**10.8%**	3.0%	25.4%	**94.6%**	94.1%	95.0%	**2.1**	0.7	5.9	**1.0**	1.0	1.0
Bleeding hemoptisis	Days 0-2	**0.2%**	0.0%	1.1%	**99.3%**	99.1%	99.5%	**1.6%**	0.0%	8.8%	**94.5%**	94.0%	95.0%	**0.3**	0.0	2.1	**1.0**	1.0	1.0
Bleeding injection site	Days 0-2	**0.0%**	0.0%	0.7%	**100.0%**	99.9%	100.0%	**0.0%**	0.0%	70.8%	**94.5%**	94.0%	95.0%	**0.0**	0.0	NA	**1.0**	1.0	1.0
Bleeding melena	Days 0-2	**1.2%**	0.4%	2.6%	**95.2%**	94.7%	95.6%	**1.4%**	0.5%	3.1%	**94.4%**	93.9%	94.9%	**0.3**	0.1	0.6	**1.0**	1.0	1.0
Bleeding other	Days 0-2	**1.8%**	0.8%	3.3%	**98.8%**	98.5%	99.0%	**7.6%**	3.5%	13.9%	**94.8%**	94.3%	95.2%	**1.5**	0.7	2.9	**1.0**	1.0	1.0
Bleeding urine	Days 0-2	**0.2%**	0.0%	1.1%	**99.2%**	99.0%	99.4%	**1.4%**	0.0%	7.7%	**94.5%**	94.0%	95.0%	**0.3**	0.0	1.8	**1.0**	1.0	1.0
Bleeding vagina	Days 0-2	**2.0%**	1.0%	3.6%	**95.8%**	95.4%	96.3%	**2.7%**	1.3%	4.9%	**94.4%**	93.9%	94.9%	**0.5**	0.3	0.9	**1.0**	1.0	1.0
Bleeding vomito negro	Days 0-2	**0.4%**	0.0%	1.4%	**99.7%**	99.5%	99.8%	**6.3%**	0.8%	20.8%	**94.5%**	94.0%	95.0%	**1.1**	0.3	4.8	**1.0**	1.0	1.0
Bleeding hematemesis	Days 0-2	**1.2%**	0.4%	2.6%	**98.0%**	97.7%	98.3%	**3.4%**	1.3%	7.3%	**94.5%**	94.0%	95.0%	**0.6**	0.3	1.4	**1.0**	1.0	1.0
Breathlessness	Days 0-2	**7.0%**	4.9%	9.5%	**92.1%**	91.6%	92.7%	**4.6%**	3.3%	6.3%	**94.8%**	94.3%	95.2%	**0.9**	0.6	1.2	**1.0**	1.0	1.0
Chest pain	Days 0-2	**12.8%**	10.0%	16.0%	**87.4%**	86.7%	88.1%	**5.2%**	4.0%	6.6%	**94.9%**	94.4%	95.4%	**1.0**	0.8	1.3	**1.0**	1.0	1.0
Coma	Days 0-2	**1.2%**	0.4%	2.5%	**98.3%**	98.0%	98.6%	**3.6%**	1.3%	7.7%	**94.8%**	94.3%	95.2%	**0.7**	0.3	1.6	**1.0**	1.0	1.0
Confusion, disorientation	Days 0-2	**0.8%**	0.2%	2.0%	**99.0%**	98.8%	99.2%	**4.0%**	1.1%	10.0%	**94.8%**	94.4%	95.2%	**0.8**	0.3	2.1	**1.0**	1.0	1.0
Conjunctivitis	Days 0-2	**6.2%**	4.3%	8.7%	**97.4%**	97.0%	97.7%	**11.5%**	8.0%	15.8%	**95.0%**	94.5%	95.4%	**2.4**	1.7	3.4	**1.0**	0.9	1.0
Cough	Days 0-2	**18.0%**	14.8%	21.6%	**72.9%**	72.0%	73.8%	**3.4%**	2.8%	4.2%	**94.3%**	93.8%	94.9%	**0.7**	0.6	0.8	**1.1**	1.1	1.2
Contact EVD case	Days 0-2	**80.2%**	76.3%	83.7%	**80.3%**	79.5%	81.1%	**17.1%**	15.6%	18.7%	**98.8%**	98.5%	99.0%	**4.1**	3.8	4.3	**0.2**	0.2	0.3
Contact funeral	Days 0-2	**37.8%**	33.3%	42.3%	**91.9%**	91.4%	92.5%	**18.2%**	15.8%	20.8%	**96.9%**	96.5%	97.2%	**4.7**	4.1	5.4	**0.7**	0.6	0.7
Contact health facility	Days 0-2	**21.5%**	17.9%	25.6%	**91.7%**	91.1%	92.2%	**10.9%**	8.9%	13.1%	**96.1%**	95.7%	96.5%	**2.6**	2.1	3.1	**0.9**	0.8	0.9
Contact informal healer	Days 0-2	**1.3%**	0.5%	2.9%	**99.4%**	99.3%	99.6%	**9.8%**	3.7%	20.2%	**95.6%**	95.2%	96.0%	**2.4**	1.0	5.4	**1.0**	1.0	1.0
Diarrhea	Days 0-2	**24.6%**	20.9%	28.5%	**63.1%**	62.2%	64.1%	**3.4%**	2.9%	4.1%	**94.0%**	93.4%	94.6%	**0.7**	0.6	0.8	**1.2**	1.1	1.3
Eyes : light sensitvity	Days 0-2	**3.5%**	2.1%	5.5%	**98.4%**	98.2%	98.7%	**10.8%**	6.6%	16.6%	**94.9%**	94.5%	95.4%	**2.2**	1.4	3.6	**1.0**	1.0	1.0
Fever	Days 0-2	**53.0%**	48.5%	57.4%	**50.1%**	49.1%	51.1%	**5.2%**	4.6%	5.9%	**95.3%**	94.7%	95.9%	**1.1**	1.0	1.2	**0.9**	0.9	1.0
Headache	Days 0-2	**50.7%**	46.3%	55.0%	**40.4%**	39.5%	41.4%	**4.2%**	3.8%	4.8%	**94.0%**	93.3%	94.7%	**0.9**	0.8	0.9	**1.2**	1.1	1.3
Hiccups	Days 0-2	**2.0%**	0.9%	3.6%	**98.1%**	97.8%	98.4%	**5.4%**	2.6%	9.7%	**94.8%**	94.4%	95.3%	**1.0**	0.6	2.0	**1.0**	1.0	1.0
Jaundice	Days 0-2	**1.6%**	0.7%	3.0%	**98.2%**	97.9%	98.4%	**4.5%**	2.0%	8.7%	**94.8%**	94.3%	95.2%	**0.9**	0.4	1.7	**1.0**	1.0	1.0
Joint pain	Days 0-2	**37.7%**	33.5%	42.0%	**67.2%**	66.3%	68.2%	**5.8%**	5.0%	6.6%	**95.3%**	94.8%	95.8%	**1.1**	1.0	1.3	**0.9**	0.9	1.0
Myalgia	Days 0-2	**29.8%**	25.9%	34.0%	**75.0%**	74.1%	75.8%	**6.0%**	5.1%	7.0%	**95.2%**	94.7%	95.7%	**1.2**	1.0	1.4	**0.9**	0.9	1.0
Other symptoms	Days 0-2	**9.9%**	7.0%	13.5%	**89.5%**	88.8%	90.2%	**4.7%**	3.3%	6.5%	**95.0%**	94.4%	95.5%	**0.9**	0.7	1.3	**1.0**	1.0	1.0
Petechia purpura	Days 0-2	**0.0%**	0.0%	0.7%	**99.9%**	99.8%	100.0%	**0.0%**	0.0%	33.6%	**94.5%**	94.0%	95.0%	**0.0**	0.0	NA	**1.0**	1.0	1.0
Rash	Days 0-2	**3.3%**	1.9%	5.2%	**97.2%**	96.9%	97.5%	**6.1%**	3.6%	9.6%	**94.8%**	94.4%	95.3%	**1.2**	0.7	1.9	**1.0**	1.0	1.0
Dysphagia	Days 0-2	**6.0%**	4.1%	8.5%	**91.9%**	91.4%	92.5%	**3.9%**	2.7%	5.5%	**94.7%**	94.3%	95.2%	**0.7**	0.5	1.1	**1.0**	1.0	1.0
Sore throat	Days 0-2	**6.1%**	4.2%	8.5%	**93.5%**	93.0%	94.0%	**4.8%**	3.3%	6.7%	**94.9%**	94.4%	95.3%	**0.9**	0.7	1.3	**1.0**	1.0	1.0
Vomiting. nausea	Days 0-2	**35.1%**	31.0%	39.4%	**50.8%**	49.8%	51.8%	**3.6%**	3.1%	4.2%	**93.7%**	93.0%	94.3%	**0.7**	0.6	0.8	**1.3**	1.2	1.4
Abdominal pain	≥ Day 3	**54.6%**	51.8%	57.3%	**42.9%**	41.9%	43.8%	**10.7%**	9.9%	11.5%	**88.3%**	87.4%	89.2%	**1.0**	0.9	1.0	**1.1**	1.0	1.1
Anorexia	≥ Day 3	**77.0%**	74.6%	79.3%	**32.8%**	31.9%	33.8%	**12.5%**	11.7%	13.2%	**92.0%**	91.1%	92.9%	**1.1**	1.1	1.2	**0.7**	0.6	0.8
Asthenia	≥ Day 3	**85.3%**	83.2%	87.2%	**27.6%**	26.7%	28.5%	**12.7%**	12.0%	13.4%	**93.8%**	92.9%	94.6%	**1.2**	1.1	1.2	**0.5**	0.5	0.6
Any bleeding	≥ Day 3	**17.2%**	15.2%	19.4%	**86.2%**	85.5%	86.8%	**13.4%**	11.7%	15.1%	**89.4%**	88.7%	90.0%	**1.2**	1.1	1.4	**1.0**	0.9	1.0
Bleeding epistaxis	≥ Day 3	**3.9%**	2.9%	5.2%	**96.4%**	96.0%	96.8%	**12.9%**	9.6%	16.7%	**88.0%**	87.4%	88.7%	**1.1**	0.8	1.5	**1.0**	1.0	1.0
Bleeding gums	≥ Day 3	**4.9%**	3.8%	6.3%	**99.6%**	99.4%	99.7%	**60.0%**	49.7%	69.7%	**88.5%**	87.8%	89.1%	**11.0**	7.4	16.3	**1.0**	0.9	1.0
Bleeding hemoptisis	≥ Day 3	**1.1%**	0.6%	1.9%	**98.7%**	98.5%	98.9%	**10.9%**	6.1%	17.7%	**88.0%**	87.3%	88.6%	**0.9**	0.5	1.6	**1.0**	1.0	1.0
Bleeding injection site	≥ Day 3	**2.9%**	2.0%	4.0%	**99.9%**	99.8%	99.9%	**77.8%**	62.9%	88.8%	**88.3%**	87.6%	88.9%	**25.6**	12.7	51.6	**1.0**	1.0	1.0
Bleeding melena	≥ Day 3	**4.9%**	3.8%	6.3%	**93.7%**	93.2%	94.2%	**9.5%**	7.4%	12.1%	**87.9%**	87.2%	88.6%	**0.8**	0.6	1.0	**1.0**	1.0	1.0
Bleeding other	≥ Day 3	**1.9%**	1.2%	2.8%	**98.9%**	98.7%	99.1%	**18.1%**	11.8%	25.9%	**88.5%**	87.9%	89.1%	**1.7**	1.1	2.6	**1.0**	1.0	1.0
Bleeding urine	≥ Day 3	**0.6%**	0.2%	1.2%	**99.0%**	98.8%	99.2%	**7.4%**	3.0%	14.6%	**88.0%**	87.3%	88.6%	**0.6**	0.3	1.3	**1.0**	1.0	1.0

Variable	Time-to-presentation	**Sensitivity** (95%CI)			**Specificity** (95%CI)			**PPV** (95%CI)			**NPV** (95%CI)			**LR+** (95%CI)			**LR-** (95%CI)		
Bleeding vagina	≥ Day 3	**2.4%**	1.6%	3.4%	**96.6%**	96.2%	97.0%	**8.8%**	5.9%	12.3%	**87.9%**	87.3%	88.6%	**0.7**	0.5	1.0	**1.0**	1.0	1.0
Bleeding vomito negro	≥ Day 3	**1.0%**	0.5%	1.7%	**99.5%**	99.3%	99.6%	**20.3%**	11.0%	32.8%	**88.0%**	87.4%	88.7%	**1.9**	1.0	3.5	**1.0**	1.0	1.0
Bleeding hematemesis	≥ Day 3	**4.5%**	3.4%	5.8%	**97.7%**	97.4%	98.0%	**21.4%**	16.5%	26.9%	**88.2%**	87.6%	88.8%	**2.0**	1.5	2.7	**1.0**	1.0	1.0
Breathlessness	≥ Day 3	**12.2%**	10.4%	14.1%	**88.0%**	87.4%	88.7%	**11.6%**	9.9%	13.5%	**88.6%**	87.9%	89.2%	**1.0**	0.9	1.2	**1.0**	1.0	1.0
Chest pain	≥ Day 3	**24.8%**	22.5%	27.3%	**81.5%**	80.7%	82.3%	**14.7%**	13.3%	16.3%	**89.4%**	88.7%	90.0%	**1.3**	1.2	1.5	**0.9**	0.9	1.0
Coma	≥ Day 3	**2.3%**	1.5%	3.2%	**98.3%**	98.0%	98.5%	**14.4%**	9.8%	20.2%	**88.6%**	87.9%	89.2%	**1.3**	0.9	1.9	**1.0**	1.0	1.0
Confusion, disorientation	≥ Day 3	**3.8%**	2.8%	5.0%	**98.3%**	98.1%	98.6%	**22.7%**	17.2%	29.0%	**88.7%**	88.1%	89.3%	**2.3**	1.6	3.1	**1.0**	1.0	1.0
Conjunctivitis	≥ Day 3	**10.3%**	8.7%	12.2%	**97.4%**	97.1%	97.7%	**34.0%**	29.3%	39.0%	**89.3%**	88.7%	89.9%	**4.0**	3.2	4.9	**0.9**	0.9	0.9
Cough	≥ Day 3	**19.2%**	17.0%	21.4%	**66.6%**	65.7%	67.5%	**6.7%**	5.9%	7.6%	**86.8%**	86.0%	87.5%	**0.6**	0.5	0.6	**1.2**	1.2	1.3
Contact EVD case	≥ Day 3	**58.8%**	55.9%	61.7%	**86.4%**	85.6%	87.0%	**33.9%**	31.8%	36.0%	**94.6%**	94.1%	95.1%	**4.3**	4.0	4.6	**0.5**	0.4	0.5
Contact funeral	≥ Day 3	**32.7%**	30.0%	35.6%	**92.9%**	92.3%	93.4%	**34.2%**	31.4%	37.1%	**92.4%**	91.9%	92.9%	**4.6**	4.1	5.1	**0.7**	0.7	0.8
Contact health facility	≥ Day 3	**30.1%**	27.4%	32.9%	**88.1%**	87.5%	88.7%	**22.4%**	20.3%	24.6%	**91.7%**	91.1%	92.3%	**2.5**	2.3	2.8	**0.8**	0.8	0.8
Contact informal healer	≥ Day 3	**3.9%**	2.9%	5.2%	**98.6%**	98.3%	98.8%	**23.9%**	17.9%	30.7%	**90.0%**	89.5%	90.6%	**2.8**	2.0	3.9	**1.0**	1.0	1.0
Diarrhea	≥ Day 3	**46.6%**	43.8%	49.3%	**63.6%**	62.6%	64.5%	**14.0%**	12.9%	15.1%	**90.3%**	89.6%	91.0%	**1.3**	1.2	1.4	**0.8**	0.8	0.9
Eyes : light sensitvity	≥ Day 3	**1.7%**	1.0%	2.6%	**98.5%**	98.2%	98.7%	**12.7%**	8.1%	18.8%	**88.5%**	87.9%	89.1%	**1.1**	0.7	1.8	**1.0**	1.0	1.0
Fever	≥ Day 3	**47.2%**	44.4%	50.0%	**50.0%**	49.0%	51.0%	**10.4%**	9.6%	11.2%	**88.5%**	87.7%	89.3%	**0.9**	0.9	1.0	**1.1**	1.0	1.1
Headache	≥ Day 3	**54.2%**	51.4%	57.0%	**36.4%**	35.5%	37.3%	**9.6%**	8.9%	10.3%	**86.5%**	85.4%	87.5%	**0.9**	0.8	0.9	**1.3**	1.2	1.3
Hiccups	≥ Day 3	**5.1%**	4.0%	6.5%	**96.8%**	96.4%	97.1%	**17.3%**	13.5%	21.5%	**88.7%**	88.1%	89.3%	**1.6**	1.2	2.1	**1.0**	1.0	1.0
Jaundice	≥ Day 3	**4.3%**	3.3%	5.6%	**96.5%**	96.2%	96.9%	**14.0%**	10.7%	17.9%	**88.6%**	88.0%	89.2%	**1.3**	0.9	1.7	**1.0**	1.0	1.0
Joint pain	≥ Day 3	**48.1%**	45.3%	50.9%	**59.9%**	59.0%	60.9%	**13.2%**	12.2%	14.2%	**90.2%**	89.4%	90.9%	**1.2**	1.1	1.3	**0.9**	0.8	0.9
Myalgia	≥ Day 3	**41.4%**	38.6%	44.2%	**68.5%**	67.6%	69.4%	**14.3%**	13.1%	15.4%	**90.2%**	89.5%	90.9%	**1.3**	1.2	1.4	**0.9**	0.8	0.9
Other symptoms	≥ Day 3	**9.7%**	7.8%	11.9%	**89.4%**	88.7%	90.1%	**10.5%**	8.5%	12.8%	**88.6%**	87.8%	89.3%	**0.9**	0.7	1.1	**1.0**	1.0	1.0
Petechia purpura	≥ Day 3	**0.4%**	0.1%	1.0%	**99.8%**	99.7%	99.9%	**26.3%**	9.1%	51.2%	**88.0%**	87.4%	88.7%	**2.6**	0.9	7.3	**1.0**	1.0	1.0
Rash	≥ Day 3	**4.3%**	3.3%	5.6%	**96.5%**	96.1%	96.8%	**13.8%**	10.5%	17.6%	**88.6%**	88.0%	89.2%	**1.2**	0.9	1.6	**1.0**	1.0	1.0
Dysphagia	≥ Day 3	**23.7%**	21.3%	26.1%	**89.5%**	88.9%	90.1%	**22.6%**	20.4%	25.0%	**90.1%**	89.5%	90.7%	**2.3**	2.0	2.5	**0.9**	0.8	0.9
Sore throat	≥ Day 3	**19.8%**	17.6%	22.1%	**92.4%**	91.8%	92.9%	**25.1%**	22.4%	27.9%	**89.9%**	89.3%	90.5%	**2.6**	2.3	3.0	**0.9**	0.8	0.9
Vomiting. nausea	≥ Day 3	**56.7%**	53.9%	59.4%	**49.4%**	48.5%	50.4%	**12.4%**	11.6%	13.3%	**90.0%**	89.2%	90.8%	**1.1**	1.1	1.2	**0.9**	0.8	0.9

Appendix Table 2. Nine versions of the algorithm applied to the testing dataset, with different predictor combinations and various individual scores.*

Predictor	Time-to-presentation	OR	Version of the algorithm and individual scores for each predictor								
			**4.1**	**4.2**	**4.3**	**4.4**	**4.5**	**4.21**	**4.22**	**4.23**	**4.24**
**Diarrhea**	Days 0-2	NS	-2	-1	0	0	-1	-1	-2	-1	-1
	≥ Day 3	1.6	+2	+1	+2	+1	+1	+1	+2	+1	+1
**Asthenia**	Days 0-2	1.9	+2	+1	+2	+1	+1	+1	+3	+1	+1
	≥ Day 3	1.9	+2	+1	+2	+1	+1	+1	+3	+1	+1
Myalgia	Days 0-2	NS	0	0	-	-	-	0	0	-	0
	≥ Day 3	1.3	+1	+1	-	-	-	+1	+1	-	+1
Anorexia	Days 0-2	NS	-	-	-	-	-	-	0	-	0
	≥ Day 3	1.7	-	-	-	-	-	-	+1	-	+1
Abdominal pain	Days 0-2	0.4	-2	-1	-2	-1	-1	-1	-3	-2	-2
	≥ Day 3	0.8	0	0	0	0	0	0	0	0	0
Headache	Days 0-2	0.7	-2	-1	-2	-1	-1	-1	-	-	-1
	≥ Day 3	0.6	-2	-1	-2	-1	-1	-1	-	-	-1
Conjunctivitis	Days 0-2	NS	+3	+1	0	0	0	+1	0	+1	+1
	≥ Day 3	2.4	+3	+1	+3	+1	+2	+1	+2	+2	+2
Sore throat	Days 0-2	NS	+2	+1	0	0	0	+1	0	0	0
	≥ Day 3	1.8	+2	+1	+3	+1	+2	+2	+3	+2	+2
Dysphagia	Days 0-2	NS	0	0	0	0	0	0	0	-	0
	≥ Day 3	1.8	+2	+1	+1	+1	+1	+2	+1	-	+1
Cough	Days 0-2	NS	0	0	0	0	0	0	0	0	0
	≥ Day 3	0.5	-3	-1	-3	-1	-1	-1	-1	-1	-1
Melena	Days 0-2	NS	0	0	0	0	0	0	-	0	0
	≥ Day 3	0.4	-4	-1	-3	-1	-1	-1	-	-1	-1
Hematemesis	Days 0-2	NS	0	0	-	-	-	0	0	-	0
	≥ Day 3	1.5	+2	+1	-	-	-	+1	+1	-	+1
Consultation at a health facility (MoH)	Days 0-2	2.1	0	0	0	0	0	0	0	0	0
	≥ Day 3	2.4	+2	+1	+3	+1	+1	+2	+2	+2	+2
Attended a funeral	Days 0-2	2.1	+3	+1	+3	+1	+1	+2	+3	+2	+2
	≥ Day 3	1.9	+2	+1	0	0	0	+1	+3	+1	+1

* The final version of the algorithm presented in the article (Figure 3) is equivalent to version 4.2. OR, Odds ratio, based on logistic regression diagnostics (training dataset); NS, not significant.

Appendix Table 3. Performance (AUROC) of nine versions of the algorithm to predict Ebola infection, for early presenters (0-2 days)*

Rank	Version	AUROC	Standard deviation	Lower value	Upper value
**1**	algo_4.21	0.7385	0.0534	0.7194	0.7576
**2**	algo_4.23	0.7300	0.0334	0.7178	0.7417
**3**	algo_4.24	0.7287	0.0432	0.7132	0.7441
**4**	algo_4.2 §	0.7136	0.0439	0.6978	0.7293
**5**	algo_4.22	0.7091	0.0530	0.6901	0.7280
**6**	algo_4.1	0.7033	0.0508	0.6851	0.7215
**7**	algo_4.5	0.6700	0.0469	0.6530	0.6865
**8**	algo_4.3	0.6645	0.0407	0.6500	0.6791
**9**	algo_4.4	0.6536	0.04239	0.6383	0.6687

* The final version of the algorithm presented in the article (Figure 2) is equivalent to version 4.2.

Appendix Table 4. Performance (AUROC) of nine versions of the algorithm to predict Ebola infection, for late presenters (≥3 days)*

Rank	Version	AUROC	Standard deviation	Lower value	Upper value
**1**	algo_4.1	**0.7446**	0.0417	0.7297	0.7595
**2**	algo_4.24	**0.7395**	0.0295	0.7290	0.7501
**3**	algo_4.21	**0.7367**	0.0334	0.7248	0.7487
**4**	algo_4.2 §	**0.7333**	0.0291	0.7229	0.7437
**5**	algo_4.22	**0.7324**	0.0352	0.7198	0.7450
**6**	algo_4.3	**0.7283**	0.0290	0.7180	0.7387
**7**	algo_4.5	**0.7218**	0.0297	0.7112	0.7324
**8**	algo_4.23	**0.7210**	0.0384	0.7072	0.7348
**9**	algo_4.4	**0.7062**	0.0242	0.6975	0.7149

* The final version of the algorithm presented in the article (Figure 2) is equivalent to version 4.2.

Appendix Table 5. Distribution of priority variables by EVD status (EVD- or EVD+). The testing population (14,346 EVD-suspected patients) is stratified in three categories: “absence of priority variables”, “absence of priority variables but at least one missing value”, “at least one priority variable”. Within the prioritized subset (“at least one priority variable”), the proportion of each of the four priority variables is shown.

Category	Proportion in EVD- stratum (%)	Proportion in EVD+ stratum (%)	Overall proportion (%)
**Absence of priority variables**	66.7% (9,361/14,027)	15.7% (50/319)	65.6% (9,411/14,346)
**Unknown (absence of priority variables but at least one missing value)**	25.2% (3,532/14,027)	31.7% (101/319)	25.3% (3,633/14,346)
**At least one priority variable**	8.1% (1,134/14,027)	52.7% (168/319)	9.1% (1,302/14,346)
**Total**	100%	100%	100%
Contact with an EVD case	*8.7% (987/11,285)*	*71.2% (153/215)*	*9.9% (1,140/11,500)*
Contact with an informal healer	*1.0% (118/12,222)*	*4.1% (9/219)*	*0.9% (127/12,441)*
Bleeding at the injection site	*0.1% (14/12,749)*	*1.0% (3/290)*	*0.1% (17/13,039)*
Bleeding gums	*0.2% (31/12,758)*	*3.4% (10/290)*	*0.3% (41/13,048)*

Appendix Table 6. Age and sex distributions among EVD-suspected patients included in the 2 prospective substudies

	Sub-study		
Category	*Beni, 2020*	*Equateur, 2020*	Total
**No strata**	2217	449	2666
**Age group**			
0-5 years	496 (20.8%)	130 (29.3%)	626 (23.6%)
6-12 years	672 (28.2%)	83 (18.7%)	755 (28.4%)
13-25 years	410 (19.7%)	62 (14.0%)	472 (17.8%)
>25 years	634 (26.6%)	169 (38.1%)	803 (30.2%)
**Sex**			
Female	1091 (49.6%)	231 (52.1%)	1322 (50.0%)
Male	1109 (50.4%)	212 (47.9%)	1321 (50.0%)
**Time-to-registration (from onset, in days)**			
0-2	885 (40.0%)	157 (34.4%)	1032 (38.8%)
3-5	964 (43.6%)	184 (42.3%)	1148 (43.2%)
≥ 6	363 (16.1%)	116 (25.4%)	479 (18.0%)
**Vaccination status upon evaluation**			
Not vaccinated	1118 (50.9%)	341 (76.1%)	1459 (55.2%)
Vaccinated without card	1062 (48.3%)	95 (21.2%)	1157 (43.7%)
Vaccinated, with card	17 (0.8%)	12 (2.7%)	29 (1.1%)

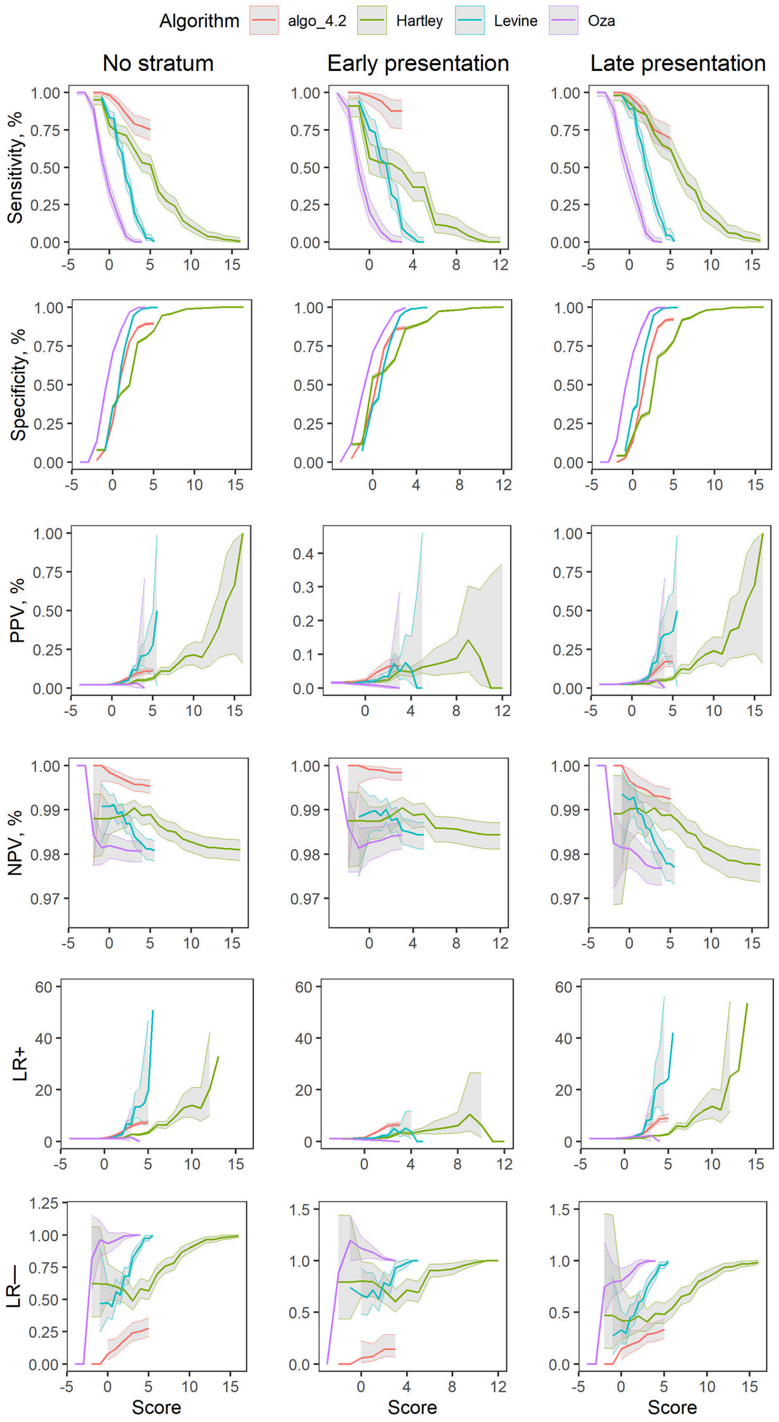

Appendix Figure 1. Diagnostic performance of the newly developed algorithm (version 4.2, all components) with those of 3 previously developed versions. All 4 algorithms were applied on the same testing population (see Methods section). Classification performance was evaluated using sensitivity, specificity, positive predictive value (PPV), negative predictive value (NPV), and likelihood ratios of positive (LR+) and negative tests (LR−).

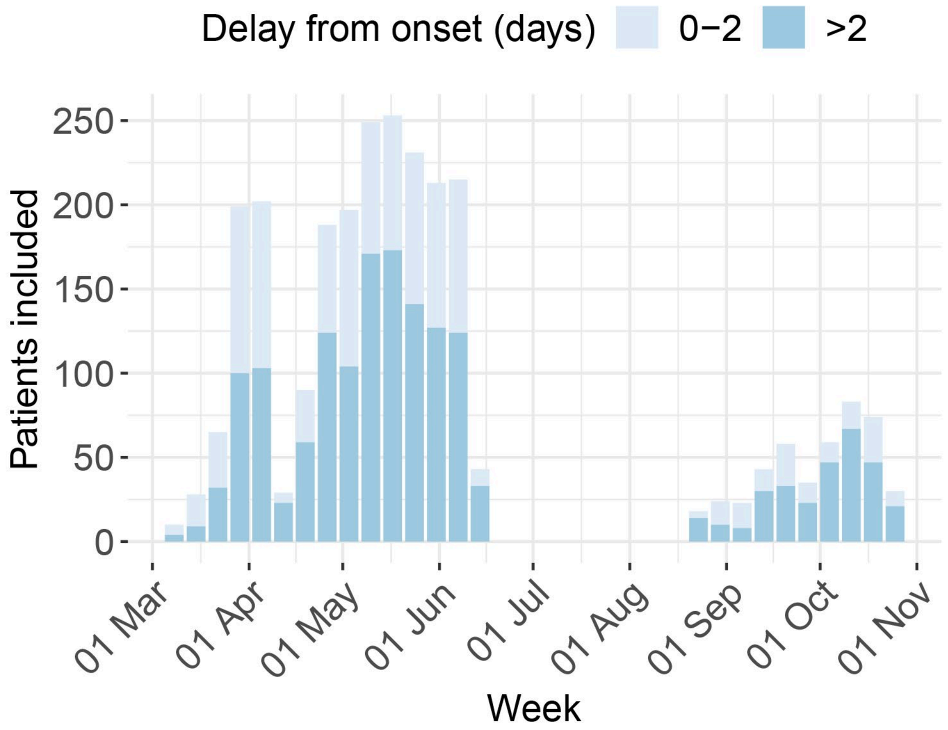

Appendix Figure 2. Inclusion dates of EVD-suspected patients in the prospective substudies (10th and 11th epidemics in the Democratic Republic of the Congo).
